# Proposed mechanisms of action participating in the hypoglycemic effect of the traditionally used *Croton guatemalensis* Lotsy and junceic acid, its main compound

**DOI:** 10.3389/fphar.2024.1436927

**Published:** 2024-10-16

**Authors:** Angelina Daniela Moreno-Vargas, Adolfo Andrade-Cetto, Fernanda Artemisa Espinoza-Hernández, Gerardo Mata-Torres

**Affiliations:** ^1^ Laboratorio de Etnofarmacología, Facultad de Ciencias, Universidad Nacional Autónoma de México, Ciudad Universitaria, Coyoacán, Mexico; ^2^ Posgrado en Ciencias Biológicas, Unidad de Posgrado, Ciudad Universitaria, Coyoacán, Mexico

**Keywords:** *Croton guatemalensis*, junceic acid, traditional medicine, type 2 diabetes, hypoglycemic, hepatic glucose production inhibition

## Abstract

*Croton guatemalensis* Lotsy (Euphorbiaceae) is an important traditional medicine that is used by the Cakchiquels of Guatemala to control hyperglycemia in patients with type 2 diabetes. Previous studies have shown that administration of this plant induces an acute hypoglycemic effect during fasting and that the main compound is junceic acid, a diterpenoid with a clerodane skeleton; however, junceic acid has not been reported to have hypoglycemic activity in the literature. As the mechanisms involved in the hypoglycemic effect of *C. guatemalensis* remain unknown, the objective of the present investigation was to elucidate the hypoglycemic mechanisms of this species, as well as its major compound, junceic acid. The results indicated that, similar to complete extract, junceic acid exhibited a hypoglycemic effect in hyperglycemic rats. Both *C. guatemalensis* extract and junceic acid inhibited the activity of two rate-limiting enzymes involved in hepatic glucose production; however, compared with chlorogenic acid, junceic acid had a more potent effect on glucose-6-phosphatase levels than chlorogenic acid, which was used as a positive control. Furthermore, both fasting and postprandial insulin levels decreased in healthy and hyperglycemic rats despite reduced blood glucose levels in both metabolic states, suggesting a potential insulin-sensitizing effect. However, neither of these compounds potentiated the effect of insulin in insulin tolerance tests nor inhibited the enzyme activity of protein tyrosine phosphatase 1B, a negative regulator of the insulin pathway. Therefore, the insulin-sensitizing effect is thought to be independent of insulin and mediated by potential activation of the AMP-activated protein kinase pathway. The specific activation of this master regulator in β-cells results in the inhibition of insulin secretion in a healthy state and the restoration of the insulin response under conditions of glucotoxicity; these effects were observed after the administration of the extract and junceic acid in healthy and hyperglycemic rats. Overall, the main findings of this study establish a basis of the mechanisms of action of *C. guatemalensis* and its main compound, junceic acid, in terms of their hypoglycemic effect.

## 1 Introduction

Poor nutrition, consumption of calorie-dense food, low physical activity, stress, and poor sleep quality are current lifestyle factors that have been associated with an increase in the incidence of chronic diseases, such as type 2 diabetes (T2D) (Niebuur et al., 2023). According to the International Diabetes Federation (IDF, 2021), there were 537 million people living with diabetes in 2021, and this number is estimated to increase by 46% by 2045. Diabetes is a chronic disease that occurs when blood glucose levels are exacerbated because the body does not produce enough insulin or because this hormone does not work effectively. The IDF recognizes four types of diabetes in accordance with their etiology, of which T2D has greater relevance as it represents approximately 90% of all diabetes cases and is related to the obesity epidemy (Peñalvo, 2024).

One of the driving factors for the development of hyperglycemia, which is characteristic of T2D, is insulin resistance (IR), which is a condition associated with an increased percentage of body fat, mainly in the abdominal area (ElSayed et al., 2024a). Depending on the tissue that presents with IR, assorted abnormalities affect glucose homeostasis under certain metabolic states. For instance, fasting hyperglycemia occurs primarily because of glucose overproduction caused by hepatic IR (mainly through the gluconeogenic pathway) (Mata-Torres et al., 2021). On the other hand, in the postprandial state, there is insufficient insulin release because of pancreatic β-cell dysfunction, which, together with IR, favors high glucose peaks after food consumption (Maffettone et al., 2018). Maintaining long-term hyperglycemia, both in the fasting and postprandial states, leads to the development of micro- and macrovascular complications that reduce the quality of life of people living with this disease (Fonseca, 2003; ElSayed et al., 2024b).

With the aim of delaying complications caused by poorly controlled hyperglycemia, health systems recommend early and personalized therapeutic intervention with hypoglycemic agents (ElSayed et al., 2024c). Currently, there is a large arsenal of drugs with specific targets that regulate glucose levels both in the fasting state and the postprandial state. However, in addition to the use of these hypoglycemic agents to control hyperglycemia, it has been reported that diabetic patients, particularly those belonging to rural communities, also resort to the use of traditional medicine to treat this disease (Cruz and Andrade-Cetto, 2015). As stated by the World Health Organization (World Health Organization, 2019), approximately 88% of the member countries of this organization use traditional and complementary medicine to maintain health and treat diseases such as T2D. Therefore, there is growing interest in describing the therapeutic effects of medicinal plants in the scientific community. In this context, by using the ethnopharmacological approach, which involves the study of the therapeutic effects of traditionally used natural products, plant-based compounds can be identified, modified, and implemented as new therapies for diabetes management (Andrade-Cetto and Heinrich, 2011; Yedjou et al., 2023).

Previously, the bark of *Croton guatemalensis* Lotsy (Euphorbiaceae), commonly known as “copalchí,” was reported to be used by the Cakchiquel community of Guatemala to treat T2D (Cruz and Andrade-Cetto, 2015). According to an ethnopharmacological study, this species was recommended for further studies due to its medicinal and cultural importance and the lack of phytochemical and pharmacological studies to support its traditional use. The hypoglycemic effect of the aqueous and ethanol-water extracts was subsequently verified by Andrade-Cetto et al. (2019) in hyperglycemic rats. In this study, the ethanol-water extract at the traditional dose of 30 mg/kg body weight (b.w.) exerted the greatest hypoglycemic effect. Moreover, although this extract could manage the postprandial hyperglycemia generated by maltose and sucrose loads, it did not inhibit the enzymatic activity of α-glucosidases isolated from the small intestine of rats. Therefore, the hypoglycemic mechanism of *C. guatemalensis* remains unknown.

Subsequent phytochemical studies revealed that the main compound in *C. guatemalensis* is junceic acid, a diterpene with a clerodane skeleton whose pharmacological effects related to diabetes have not been reported in the literature; furthermore, rutin and epicatechin, two flavonoids that have been shown to have an insulin secretagogue effect, were also identified (Escandón-Rivera et al., 2022; Wen et al., 2022).

To elucidate the hypoglycemic mechanism of *C. guatemalensis*, assess the potential contribution of junceic acid to the hypoglycemic effect, and provide new information on the ethnopharmacological effects of the genus (Espinoza-Hernández et al., 2023), the acute hypoglycemic effect of junceic acid, as well as the ethanol-water extract, was tested *in vivo*. Afterward, as the extract was shown to exert a significant effect on fasting hyperglycemia in previous studies, the main mechanism involved was evaluated *in vivo* and *in vitro*, namely, the inhibition of hepatic glucose production. In addition, the effect of the extract and junceic acid on insulin levels under fasting and postprandial conditions was assessed with the aim of providing a more detailed overview of their biological actions in both metabolic states and guiding future experiments on their mechanisms of action. Finally, the potential effect on insulin sensitivity was investigated *in vivo* and *in vitro*.

## 2 Materials and methods

### 2.1 Chemicals and reagents

Streptozotocin (STZ; S0130), nicotinamide (NA; N0636), glucose (G7021), sodium pyruvate (P2256), sucrose (S7903), ethylenediaminetetraacetic acid (EDTA; EDS), 4-(2-hydroxyethyl)-1-piperazineethanesulfonic acid (HEPES; H3375), imidazole (I0250), chlorogenic acid (C3878), glucose-6-phosphate (G6P; A2252), ascorbic acid (A7506), malachite green (M6880), MgCl_2_ (M9272), TWEEN^®^ 20 (P1379), sodium dodecyl sulfate (SDS; L3771), Tris-HCl (T3253), fructose-1,6-bisphosphate (FBP; F6803), adenosine 5′-monophosphate monohydrate (AMP; A2252), protease inhibitor cocktail tablets (11836153001), ethylene glycol-bis(2-aminoethylether)-*N,N,N′,N′*-tetraacetic acid (EGTA; E4378), DL–dithiothreitol (DTT; D0632), β-mercaptoethanol (M6250), Na_3_VO_4_ (S6508), and *p*-nitrophenyl phosphate (pNPP; P4744) were purchased from Sigma-Aldrich (Germany). Ammonium molybdate (AT0330-5) was bought from Tecsiquim (Mexico). NaCl (3624-01) was acquired from J.T. Baker (United States). NP-40 (492,016) was obtained from Merck-Millipore (Germany). Metformin, glibenclamide, repaglinide, pioglitazone, and isophane insulin (Aurax^®^) were purchased from a local pharmacy.

### 2.2 Plant collection

**FIGURE 1:**
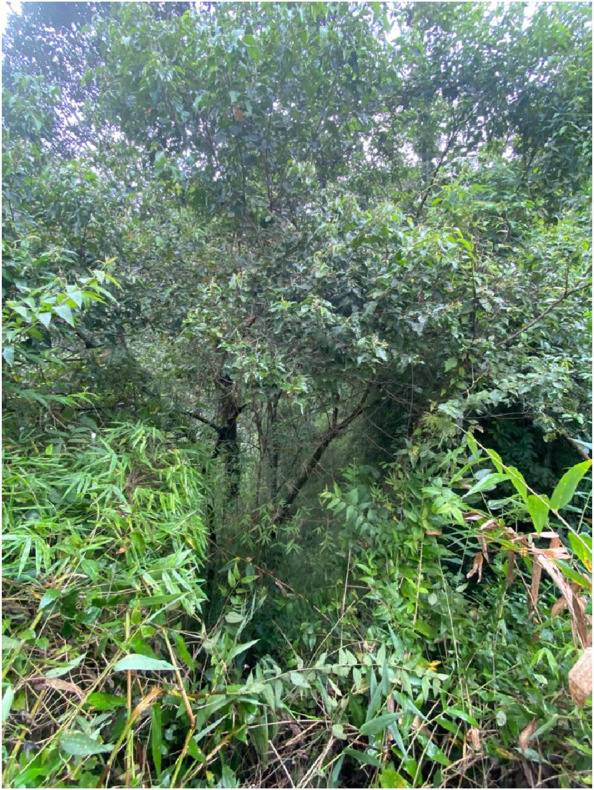
Photograph of *C. guatemalensis* at the collection site.

The bark of *C. guatemalensis* was collected in the Department of Chimaltenango with the help of Carola Cruz and Dr. Jorge Mario Vargas from “Universidad de San Carlos,” which is located 56 km west of Guatemala City at 1,817 m. a.s.l. at 14° 39′38″ N and 90° 49′10″ (Figure 1). A specimen was deposited at the Deshidrafarmy-Farmaya Herbarium (voucher CFEH 1259). The plant material collected and used to prepare the extract tested in the present study was the same utilized by Andrade-Cetto et al. (2019) and Escandón-Rivera et al. (2022).

### 2.3 Preparation of the extract and obtention of the major compound

An ethanol-water extract (EtOH-WCg) was prepared according to the methodology proposed by Andrade-Cetto et al. (2019) by placing 20 g of the dried and ground bark in 500 mL of a 1:1 mixture of water and ethanol at 40°C with constant stirring for 4 h (two extractions were carried out). Both extractions were mixed and subsequently filtered, distilled, and lyophilized to obtain the dry extract. Junceic acid was isolated from EtOH-WCg and provided by Dr. Sonia Escandón-Rivera, following the protocol described in Escandón-Rivera et al. (2022).

The dose of the extract used to perform the *in vivo* experiments was 30 mg/kg b. w., which was previously calculated considering the traditional use (Andrade-Cetto et al., 2019). On the other hand, a dose of 5.6 mg/kg b. w. of junceic acid was calculated from the yield obtained from the initial 20 g of the bark to prepare the extract (Escandón-Rivera et al., 2022), which is the amount traditionally consumed by a 70-kg person (Cruz and Andrade-Cetto, 2015). The phytochemical profile of the tested extract can be seen at doi: 10.3390/plants11223159.

### 2.4 *In vivo* experiments

#### 2.4.1 Experimental animals

All procedures carried out on animals were approved by the Academic Ethics and Scientific Responsibility Commission (CEARC) of the School of Sciences, UNAM with protocol numbers PI_2021_08_23, PI_2022_21_05b, and PI_2022_22_07. Wistar rats of both sexes, 8 weeks old, were acquired and maintained under standard bioterium conditions (25°C, 55% humidity and 12:12 h light and dark photoperiod) with water and food *ad libitum*. Treatment administration (vehicle and samples) was performed orally with the help of an esophageal cannula. Blood samples were obtained from the tail vein and glucose measurements were performed in duplicate using Accu-Chek^®^ Active glucometers.

#### 2.4.2 Sample size calculation

To calculate the number of rats per group for each experiment, data obtained from pilot studies were used, applying the following formula (Charan and Biswas, 2013):

$$Sample\;size=\frac{2{SD}^{2}{\left(Z_{\alpha /2}+Z_{\beta }\right)}^{2}}{d^{2}}$$

SD = standard deviation of previous studies. $Z_{\alpha /2}$ = 1.96 (type 1 error of 5%)

$Z_{\beta }$ = 0.842 (80% power)d (effect size) = difference between means of pilot studies.

This calculation considers the 4R in Ethnopharmacology: reduce, refine, replace, and responsibility when using animal models.

#### 2.4.3 Induction of hyperglycemia

The STZ-NA-induced hyperglycemia model was selected as an animal model to perform subsequent *in vivo* experiments. Although it lacks insulin resistance, it is still a good model for evaluating the pharmacological effects of potential hypoglycemic agents because it develops characteristics like human T2D, such as stable and moderate hyperglycemia, moderate reduction of pancreatic β cells and insulin reserves, impaired glucose tolerance, impaired glucose-stimulated insulin secretion, and classic symptoms of diabetes (Masiello et al., 1998). Furthermore, this animal model mimics virtually altered insulin sensitivity due to a lower pancreatic insulin reserve and provides responsiveness to insulin secretagogues, making it a useful tool to test agents with insulinotropic capacity potentially useful in the treatment of T2D (Fröde and Medeiros, 2008; Asrafuzzaman et al., 2017).

In summary hyperglycemia was induced as described by Masiello et al. (1998), rats fasted for 12 h were administered intraperitoneally with 150 mg/kg b. w. of NA and, 15 min later, 65 mg/kg b. w. of STZ was injected intravenously. After 2 weeks, animals with blood glucose values above 180 mg/dL were selected to perform the tests described below.

#### 2.4.4 Evaluation of the hypoglycemic effect

Basal glucose levels were assessed before giving the treatments. Immediately after the administration of treatments, glucose levels were monitored every hour for 3 h (Espinoza-Hernández et al., 2021). Thirty rats fasted for 4 h were divided into five groups (n = 6 each group): 1. Normoglycemic Control (**N**), administered with physiological solution (vehicle); 2. Hyperglycemic Control (**H**), which received physiological solution; 3. Hyperglycemic Control + glibenclamide, which was administered with 5 mg/kg b. w. (Aurax^®^) (**HGli**) (Berridge et al., 1992); 4. Hyperglycemic Group + EtOH-WCg, which received 30 mg/kg b. w. (**HCg**); and 5. Hyperglycemic Group + junceic acid, administered with 5.6 mg/kg b. w. (**HJa**).

#### 2.4.5 Evaluation of the inhibition of hepatic glucose production

Rats fasted for 18 h exhibiting glucose levels between 170 and 220 mg/dL were selected. After basal glucose measurement, animals received their corresponding treatment. Fifteen minutes later, rats were injected intraperitoneally with a pyruvate solution (2 g/kg b. w.). In total, 30 rats were divided into five groups (n = 6 each group): 1. Normoglycemic Control + pyruvate (**NP**), receiving physiological solution; 2. Hyperglycemic Control + pyruvate (**HP**), which was administered with physiological solution; 3. Hyperglycemic Control + pyruvate + metformin (Aurax^®^), which was given 500 mg/kg b. w. (**HMP**); 4. Hyperglycemic Group + pyruvate + EtOH-WCg, administered with 30 mg/kg b. w. (**HCgP**); and 5. Hyperglycemic Group + pyruvate + junceic acid, which received 5.6 mg/kg b. w. (**HJaP**). After intraperitoneal injection, glucose levels were measured every 30 min for 2 h (Mata-Torres et al., 2020).

#### 2.4.6 Evaluation of fasting and postprandial insulin levels in normoglycemic and hyperglycemic animals

Normoglycemic (healthy) and hyperglycemic rats were used after 12 h of fasting selecting those with blood glucose levels between 90 and 110 mg/dL and 180–220 mg/dL, respectively. Before the administration of the treatment and every 30 min for 2 h, glucose levels were determined and additional blood samples (approximately 300 µL) were obtained, which were immediately centrifuged at 10,000 g for 10 min. Plasma obtained was kept at −40°C until further analysis. Insulin quantification was performed using the immunoassay Rat/Mouse Insulin ELISA kit EZRMI-13KEMD Millipore/Merck (Espinoza-Hernández et al., 2021).

##### 2.4.6.1 Fasting assessment

Both normoglycemic and hyperglycemic rats were divided into four groups for each condition (n = 6 each group), namely, a total of 48 rats into eight groups: Control, administered with physiological solution (**N** and **H**); Glibenclamide (Aurax^®^), which received 5 mg/kg b. w. (**NGli** and **HGli**); EtOH-WCg, which were given 30 mg/kg b. w. (**NCg** and **HCg**); and Junceic acid, receiving 5.6 mg/kg b. w. (**NJa** and **HJa**).

##### 2.4.6.2 Postprandial assessment

Both normoglycemic and hyperglycemic rats were divided into four groups for each condition (n = 6 each group) using a total of 48 rats assigned into eight groups: Control + Glucose, which were administered with physiological solution (**NG** and **HG**); Repaglinide (Prandin^®^) + Glucose, receiving 1 mg/kg b. w. (**NRepG** and **HRepG**); EtOH-WCg + Glucose, which were given 30 mg/kg b. w. (**NCgG** and **HCgG**); and Junceic acid + Glucose, administered with 5.6 mg/kg b. w. (**NJaG** and **HJaG**). A glucose load (2 g/kg b. w.) was given 5 min after the administration of each treatment.

#### 2.4.7 Insulin sensitivity assessment

Normoglycemic (healthy) rats were used after 4 h of fasting exhibiting glucose levels between 100 and 130 mg/dL. First, basal glucose was determined and, immediately after, treatments were given. Forty-5 min later, glucose levels were measured and then isophane insulin (Aurax^®^; 0.5 U/kg b. w.) was administered intraperitoneally. Glucose levels were monitored at minutes 15, 30, 60, and 120 after insulin injection. In total, 24 rats were divided into four groups (n = 6 each group): 1. Control, which received physiological solution (**C**); 2. Pioglitazone (Aurax^®^), administered with 20 mg/kg b. w. (**Pio**); 3. EtOH-WCg, which was given 30 mg/kg b. w. (**Cg**); and 4. Junceic acid, receiving 5.6 mg/kg b. w. (**Ja**) (Andrade-Cetto et al., 2024).

### 2.5 *In vitro* experiments

#### 2.5.1 Obtention of gluconeogenic regulatory enzymes

Livers were removed from 18-h fasted healthy rats (3), which were previously anesthetized with 5% isoflurane (Unit for Laboratory Animal Medicine, 2023). Then, they were homogenized in buffer (250 mM sucrose, 1 mM EDTA, 5 mM HEPES, pH 7.4), filtered, and subsequently subjected to differential centrifugation as described by Andrade-Cetto and Cárdenas-Vázquez (2010). Both the microsome-enriched supernatant (containing the enzyme complex glucose-6-phosphatase) and the cytosolic supernatant (containing the enzyme fructose-1,6-bisphosphatase) were collected and stored at −40°C until use.

#### 2.5.2 Glucose-6-phosphatase (G6Pase) enzyme activity inhibition assay

Colorimetric assays were carried out to detect the formation of a phosphomolybdate complex, which is proportional to the activity of the G6Pase enzyme complex. The reaction turns blue when G6Pase releases the inorganic phosphate and therefore the lower the color intensity, the greater the inhibition (Arion, 1989). Three independent assays were performed in triplicate following the protocol proposed by Mata-Torres et al. (2020). First, dilutions of the supernatant enriched with microsomes were prepared with buffer (250 mM sucrose, 40 mM imidazole, pH 7.0) to which the inhibitor samples were added at different concentrations (chlorogenic acid, EtOH-WCg, or junceic acid). These reaction mixtures were incubated for 5 min at 22°C. The reaction was started by adding the substrate (20 mM G6P) and incubated for 20 min at 22°C. At the end of the incubation period, stop solution was added (0.42% ammonium molybdate in 1 N H_2_SO_4_, 10% SDS, and 10% ascorbic acid) and after that the mixture was incubated for 20 min at 45°C. Finally, the absorbance was measured at 830 nm.

#### 2.5.3 Fructose-1,6-bisphosphatase (FBPase) enzyme activity inhibition assay

Colorimetric assays were performed to detect the formation of a phosphomolybdate-malachite green complex with the inorganic phosphate released by the enzyme FBPase. The reaction turns green when the complex is formed; thus, the lower the color intensity, the greater the inhibition (Baykov et al., 1988). Three independent assays were made by triplicate, following the protocol of Espinoza-Hernández et al. (2021). Before carrying out the assays, the dye solution was prepared (at least 1 day before), which contained 0.12% of malachite green dissolved in a solution of distilled water and H_2_SO_4_ (5:1). On the day of the assay, one volume of 7.5% ammonium molybdate was added to four volumes of the dye solution plus 0.17% of TWEEN^®^ 20.

The assays were carried out with substrate-enriched buffer (5 µM EDTA, 5 mM MgCl_2_, 50 mM Tris-HCl, 0.1 mM FBP, pH 7.2) to which the inhibitors were added at different concentrations (AMP, EtOH-WCg, or junceic acid). The reaction was started by adding the cytosolic supernatant previously diluted in buffer without substrate (1:9). The mixture was incubated for 20 min at 24°C and stopped by adding the dye solution. Afterwards, a second incubation was made under the previous conditions. Finally, the absorbance was measured at 630 nm.

#### 2.5.4 Obtention of protein tyrosine phosphatase 1B (PTP-1B) enzyme

One day before the isolation of the enzyme-containing subcellular fraction, lysis buffer was prepared (50 mM HEPES, 0.1 mM EGTA, 0.1 mM EDTA, 120 mM NaCl, 0.5% NP-40, pH 7.5) and enriched with one protease inhibitor cocktail tablet per 10 mL of buffer. The liver from an overnight-fasted healthy rat, previously anesthetized with isoflurane, was removed. The organ was then homogenized in lysis buffer as described by Li et al. (2010). The homogenate was filtered and subsequently centrifuged at 14,000 g for 15 min at 4°C. Supernatants were collected and stored at −40°C until use.

#### 2.5.5 Protein tyrosine phosphatase 1B (PTP-1B) enzyme activity inhibition assay

Colorimetric assays were carried out to detect the dephosphorylation of pNPP, which is proportional to the activity of the PTP-1B enzyme. Three independent assays were performed in triplicate following the protocols proposed by Guo et al. (2015) and Song et al. (2017) with slight modifications. Firstly, an assay buffer (25 mM Tris-HCl, 2 mM β-mercaptoethanol, 1 mM EDTA, 1 mM DTT, pH 7.5) was added to the reaction mixture. Then, inhibitor samples at different concentrations (Na_3_VO_4_, EtOH-WCg, or junceic acid) and enzyme supernatant were added. Afterwards, an incubation of 5 min at 37°C was made. The reaction was started by adding the substrate (10 mM pNPP) and incubated for 30 min at 37°C. Finally, the absorbance was measured at 405 nm.

### 2.6 Statistical analysis

Data are presented as mean ± standard error of the mean (SEM). All graphs and analysis were made in GraphPad Prism version 9, GraphPad Software, San Diego, California United States. For *in vivo* experiments, data were tested for normality and subsequently log-transformed as necessary. For the experiments on hypoglycemic effect and inhibition of hepatic glucose production, one-way ANOVA followed by Tukey *post hoc* tests were performed for intergroup analysis, while repeated measures ANOVA followed by Dunnett *post hoc* tests were applied for intragroup analysis. Differences detected with *p* < 0.05 were considered statistically significant. The areas under the curve (AUC) were also calculated and compared through one-way ANOVA followed by Tukey *post hoc* tests. To analyze the effect of the treatments on glucose and insulin levels in fasting and postprandial state, raw values were transformed into percentages:

$$Glucose/Insulin\;variation\;\left(\%\right)=\frac{{Glucose/Insulin}_{Tx}\times 100\%}{{Glucose/Insulin}_{T0}}-100$$

Where Tx is the value of glucose or insulin at each time and T0 is the baseline value. Next, AUC were calculated and compared by applying t tests for intergroup comparisons and repeated measures ANOVA followed by Dunnett *post hoc* tests for intragroup comparisons. For insulin tolerance tests, paired t tests were performed to compare basal time (−45 min) against T0 in the same group to assess the hypoglycemic effect before insulin intervention. In addition, the inverse AUC was obtained and compared through one-way ANOVA followed by Tukey *post hoc* tests, and the rate of glucose disappearance constant (K_ITT_) was calculated as follows:

$$K_{ITT}=Slope\left[\left({\mathrm{Ln}\;Glc}_{T0}\right),\left({\mathrm{Ln}\;Glc}_{T15}\right){,(\mathrm{Ln}\;Glc}_{T30})\right]]\times -100$$

where Ln Glc_T0_ is the natural logarithm of the glucose value in T0, Ln Glc_T15_ is the natural logarithm of the glucose value in T15, and Ln Glc_T30_ is the natural logarithm of the glucose value in T30.

Finally, for *in vitro* experiments, absorbance values were transformed into activity percentages following the next formula:

$$Enzyme\;activity\;\left(\%\right)=\frac{A_{S}-A_{SB}}{A_{C}-A_{CB}}\times 100$$

Where *A*_*S*_ is the absorbance of the inhibitor sample at a specific concentration, *A*_*SB*_ is the blank of the inhibitor sample, *A*_*C*_ is the highest absorbance (without inhibitor), and *A*_*CB*_ is the blank of the highest absorbance. Afterward, percentages were plotted on concentration-response curves to find the best non-linear regression model (three or four parameters) used to calculate the inhibitory concentration 50 (IC_50_) of each inhibitor sample.

## 3 Results

### 3.1 Hypoglycemic effect

**FIGURE 2:**
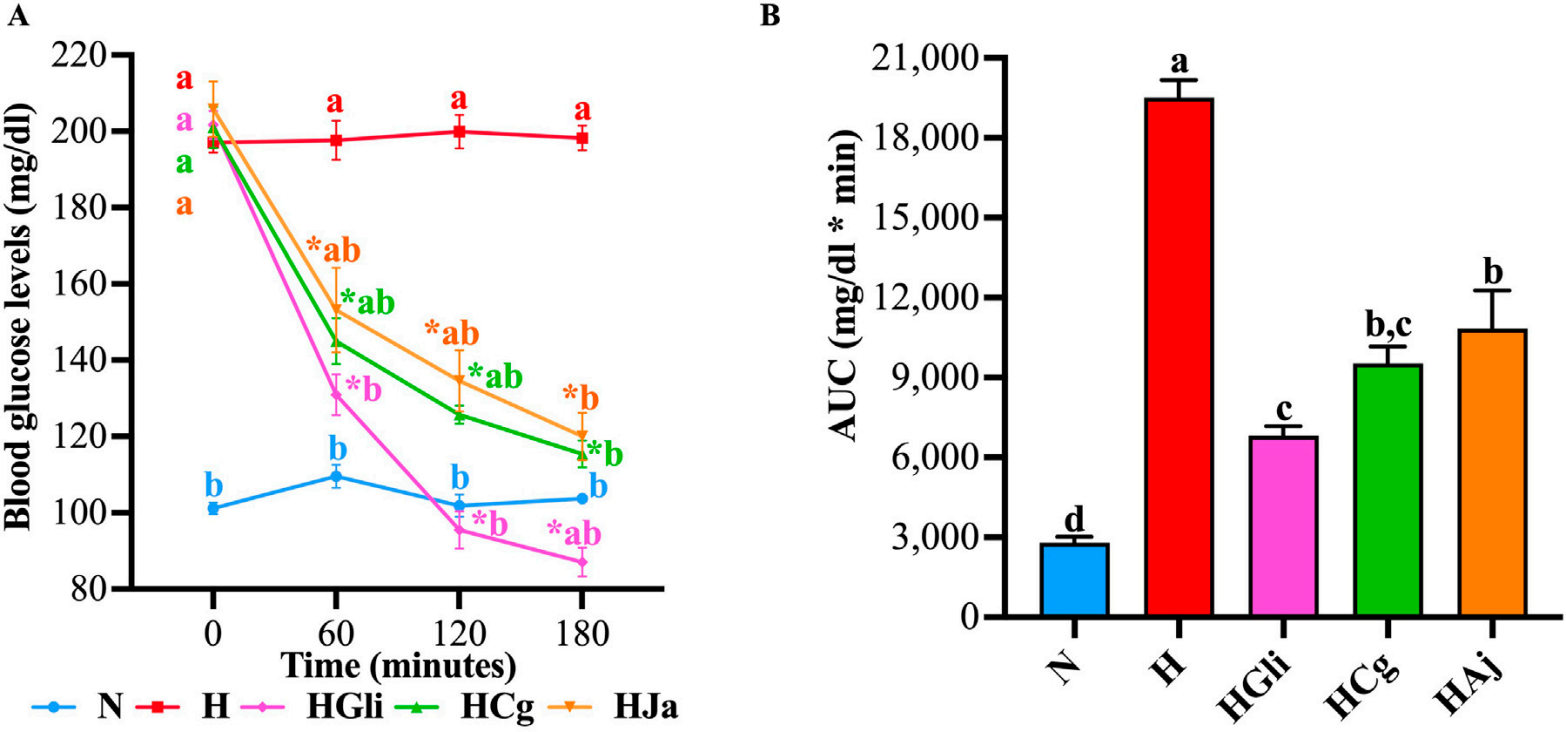
Hypoglycemic effect of EtOH-WCg and junceic acid. **(A)** Blood glucose curves in acute tests. *indicates statistically significant differences versus its initial time, ^**a**^indicates statistically significant differences versus **N**, ^**b**^indicates statistically significant differences versus **H;** *p* < 0.05. **(B)** AUC values. Different letters over the bars indicate statistically significant differences (a > b > c > d); *p* < 0.05. The values represent the mean ± SEM (n = 6). **N**, normoglycemic group; **H**, hyperglycemic group; **HGli**, hyperglycemic + glibenclamide group; **HCg**, hyperglycemic + EtOH-WCg; **HJa**, hyperglycemic + junceic acid isolated from EtOH-WCg.

To verify that the bark cortex used by Andrade-Cetto et al. (2019) maintained its hypoglycemic effect, temporal glucose curves were performed in STZ-NA-hyperglycemic rats. In these curves, it was observed that glibenclamide (**HGli**), used as a control drug, EtOH-WCg (**HCg**), and junceic acid (**HJa**) exerted a significant hypoglycemic effect 60 min after administration when compared with the hyperglycemic control group (**H**). This effect was maintained throughout the entire experiment; however, glibenclamide exhibited the greatest glucose decrease by 57% at the end of the study, EtOH-WCg and junceic acid similarly reduced hyperglycemia by 43% and 42%, respectively (Figure 2A). According to the AUC analysis, glibenclamide decreased total blood glucose by 65% compared with control (**H**), EtOH-WCg by 51%, and junceic acid by 44%. Interestingly, although all groups were different from the hyperglycemic control, the group administered with EtOH-WCg was statistically similar to the group given glibenclamide (Figure 2B). In addition, it was proven that junceic acid, the main compound of EtOH-WCg, has a glucose-lowering effect like that of the complete extract.

### 3.2 Effect on hepatic glucose production

#### 3.2.1 Pyruvate tolerance tests

**FIGURE 3:**
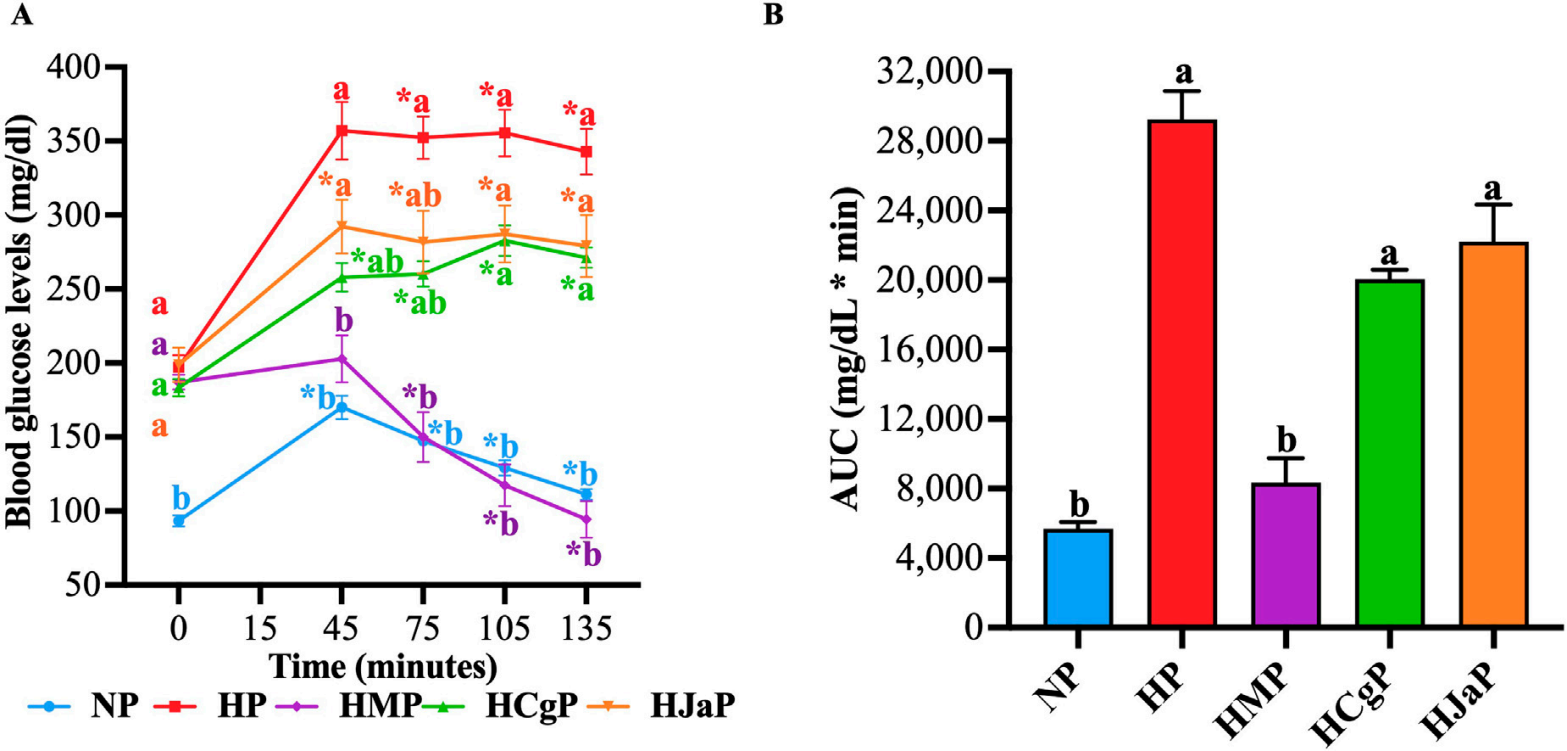
Effect of EtOH-WCg and junceic acid on hepatic glucose production. **(A)** Blood glucose levels in pyruvate tolerance tests. *Indicates statistically significant differences versus its initial time, ^**a**^indicates statistically significant differences versus **NP**, ^**b**^indicates statistically significant differences versus **HP;** *p* < 0.05. **(B)** AUC values. Different letters over the bars indicate statistically significant differences (a > b); *p* < 0.05. The values represent the mean ± SEM (n = 6). **NP**, normoglycemic + pyruvate group; **HP**, hyperglycemic + pyruvate group; **HMP**, hyperglycemic + metformin + pyruvate group; **HCgP**, hyperglycemic + EtOH-WCg + pyruvate; **HJaP**, hyperglycemic + junceic acid isolated from EtOH-WCg.

The main mechanism generating fasting hyperglycemia in T2D is hepatic glucose production and both EtOH-WCg and junceic acid showed a significant hypoglycemic effect in this state, the next objective was to corroborate if both can reduce hyperglycemia through the inhibition of this mechanism. To achieve this goal, pyruvate tolerance tests were carried out in STZ-NA-hyperglycemic rats fasted for 18 h. The results showed that in the group administered with metformin (**HMP**), used as a control drug, a significant anti-hyperglycemic effect was obtained. The hypoglycemic agent completely inhibited the hyperglycemic peak presented 30 min after pyruvate injection and later it decreased blood glucose, reaching normoglycemic levels at the end of the test. On the other hand, both EtOH-WCg (**HCgP**) and junceic acid (**HJaP**) decreased the hyperglycemic peak at 30 min by 40% and 34%, respectively, compared with the hyperglycemic control (**HP**). Afterwards, both tended to maintain lower glucose levels than those observed in **HP** throughout the experiment (Figure 3A). According to the AUC analysis, metformin decreased total blood glucose by 71% compared with control, EtOH-WCg by 31%, and junceic acid by 24% (Figure 3B). Altogether, these findings suggest that the extract and its main compound can decrease hepatic glucose production *in vivo*. However, the complete extract showed a slightly better effect than junceic acid as observed during the first hour of the experiment after pyruvate administration.

#### 3.2.2 Concentration-response inhibition assays of gluconeogenic regulatory enzymes (G6Pase and FBPase)

**FIGURE 4:**
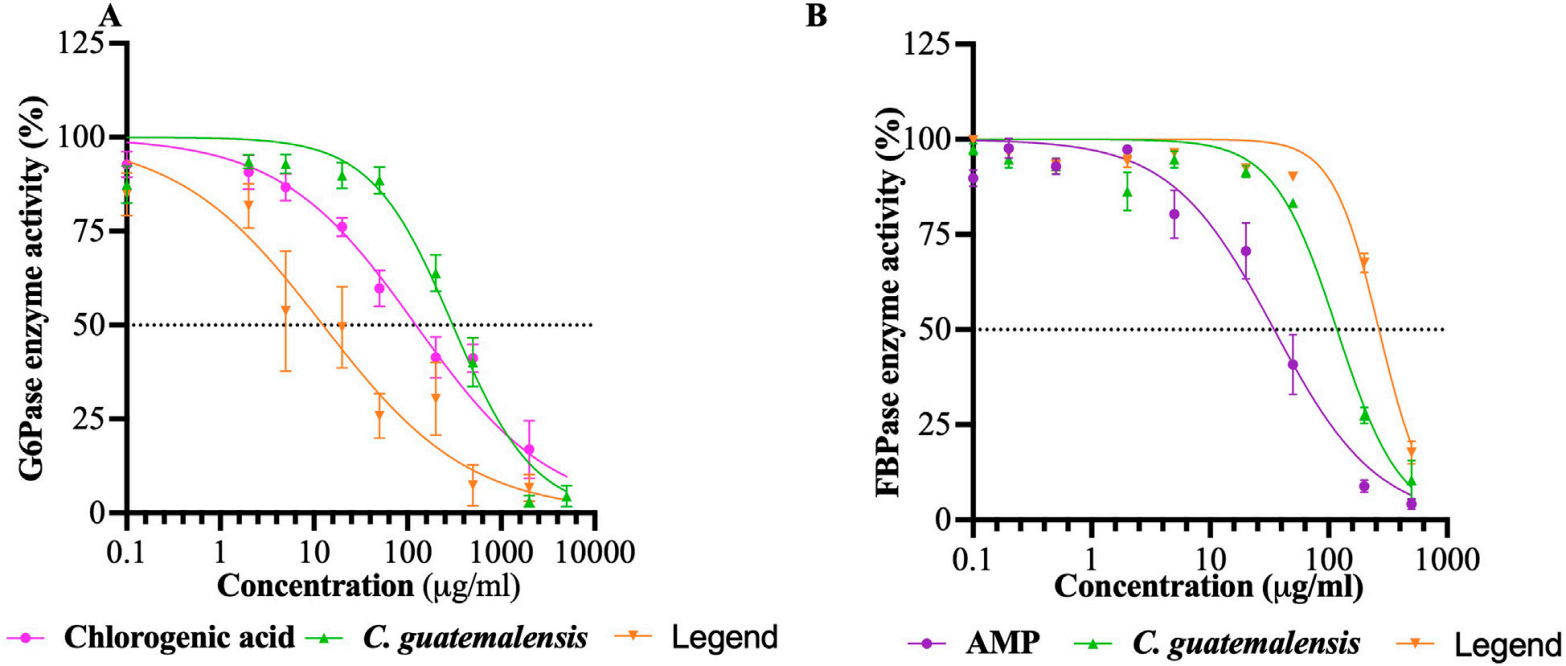
Concentration-response inhibition curves of EtOH-WCg and junceic acid on rate-limiting enzymes of hepatic glucose production. **(A)** Glucose-6-phosphatase enzyme (G6Pase). **(B)** Fructose-1,6-bisphosphatase enzyme (FBPase). Each point represents the mean ± SEM of three independent assays in triplicate.

To delve into possible specific pharmacological targets related to the inhibition of hepatic glucose production, the inhibitory capacity of EtOH-WCg and junceic acid on two relevant rate-limiting enzymes was evaluated: G6Pase, which regulates glucose output from both gluconeogenesis and glycogenolysis, and FBPase, which controls the entry of all substrates into the gluconeogenic pathway. The results revealed that the extract effectively inhibited the G6Pase activity by 97%, exhibiting the highest percentage of inhibition (Table 1; Figure 4). Regarding FBPase, EtOH-WCg inhibited the enzyme activity by 89%. Surprisingly, junceic acid was more effective and potent than chlorogenic acid in inhibiting G6Pase activity, displaying an inhibition percentage of 93% and an IC_50_ value of 18.8 μg/mL, which was 6.5 times lower than control. Regarding the FBPase activity, it showed an inhibition percentage of 82%. These outcomes indicate that direct inhibition of the enzymatic activity of both enzymes are involved in the glucose-lowering effect of fasting hyperglycemia of both EtOH-WCg and junceic acid by inhibiting hepatic glucose production. Specifically, it can be concluded that one of the primary targets of junceic acid is G6Pase, which participates not only in gluconeogenesis, but also in glycogenolysis.

**TABLE 1:** *In vitro* assessment of EtOH-WCg and junceic acid on rate-limiting enzymes of hepatic glucose production.

Glucose-6-phosphatase enzyme (G6Pase)			Fructose-1,6-bisphosphatase enzyme (FBPase)		
Sample	Inhibition %	IC_50_ ± SEM (µg/mL)	Sample	Inhibition %	IC_50_ ± SEM (µg/mL)
Clorogenic acid^a^	83	122.7 ± 21.7	AMP^a^	96	34.7 ± 4.2
EtOH-WCg	97	304.2 ± 33.5	EtOH-WCg	89	117.5 ± 8.9
Junceic acid	93	18.8 ± 5.2	Junceic acid	82	265.7 ± 10.2

^a^ Positive control.

### 3.3 Effect on fasting and postprandial insulin levels

#### 3.3.1 Fasting condition

**FIGURE 5:**
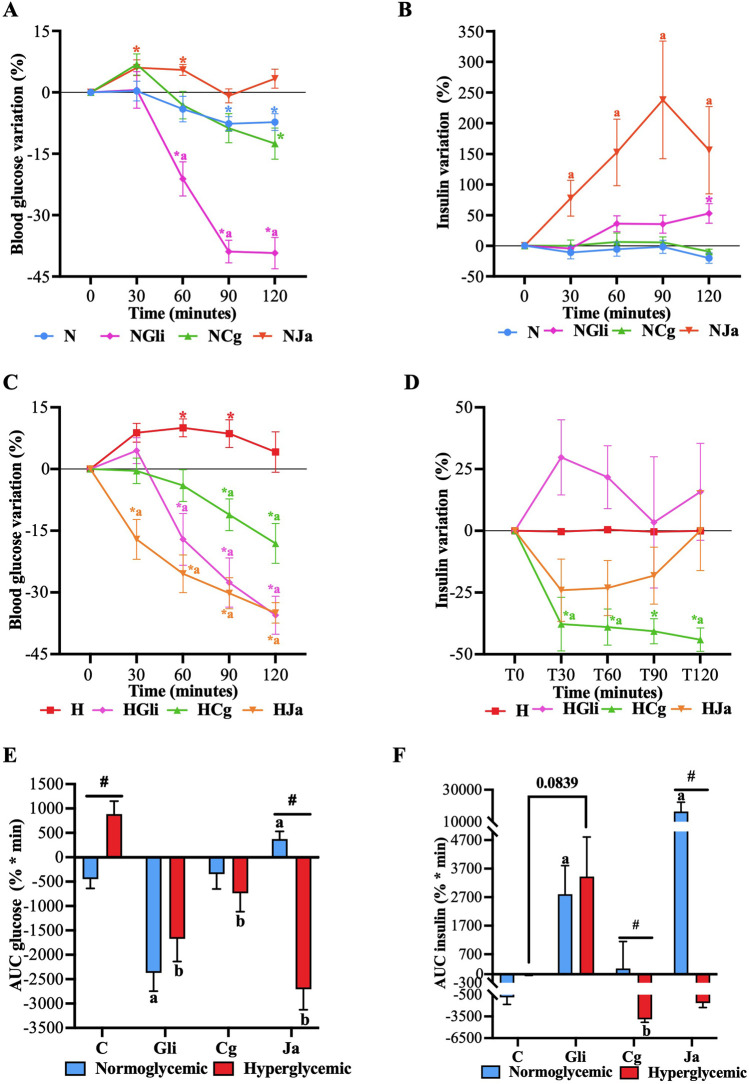
Effect of EtOH-WCg and junceic acid on insulin levels in fasting. **(A)** Blood glucose variation in normoglycemic groups; **(B)** Plasma insulin variation in normoglycemic groups; **(C)** Blood glucose variation in hyperglycemic groups; **(D)** Plasma insulin variation in hyperglycemic groups; **(E)** AUC of glucose variation; **(F)** AUC of insulin variation. *indicates statistically significant differences versus its initial time; *p* < 0.05, ^**#**^indicates statistically significant differences between normoglycemic and hyperglycemic groups per treatment; *p* < 0.05, ^**a**^indicates statistically significant differences versus **N** (only within normoglycemic groups); *p* < 0.05. ^**b**^indicates statistically significant differences versus **H** (only within hyperglycemic groups); *p* < 0.05. **N**, normoglycemic; **NGli**, normoglycemic + glibenclamide; **NCg**, normoglycemic + EtOH-WCg; **NJa**, normoglycemic + junceic acid isolated from EtOH-WCg. **H**, hiperglycemic; **HGli**, hyperglycemic + glibenclamide; **HCg**, hyperglycemic + ethanol-water extract of EtOH-WCg bark; **HJa**, hyperglycemic + junceic acid isolated from EtOH-WCg.

The role of plasma insulin in the effect of EtOH-WCg and junceic acid on the homeostatic regulation of fasting glucose in a healthy state and a hyperglycemic state was then evaluated by quantifying both glucose and insulin in normoglycemic and STZ-NA-hyperglycemic rats fasted for 12 h. Figures 5A, B showed that whilst the normal behavior of glucose in a healthy state was to decrease over time (no more than approximately 8%), insulin tended to remain stable (**N**). In contrast, glucose increased significantly without altering insulin levels in a hyperglycemic state (**H**) (Figures 5C, D). Groups that received glibenclamide (**NGli** and **HGli**) presented a significant hypoglycemic effect starting 60 min after drug administration, which was more pronounced in healthy rats (Figure 5E). This glucose-lowering effect can be explained by the increase in plasma insulin. Nevertheless, although the total insulin secreted over time by the drug was similar in both groups (Figure 5F), the moment in which it appeared in the bloodstream was different: the secretion observed in healthy rats increased over time (exhibiting the highest percentage at 120 min), whereas most of the insulin secreted in hyperglycemic rats was noted during the first 30 min with a subsequent decrease in its concentration.

Despite a hypoglycemic effect being observed in both groups administered with extract (**NCg** and **HCg**), a significantly more evident effect was promoted starting 90 min after extract administration in hyperglycemic rats. On the other hand, the glucose-lowering effect displayed in healthy rats was more marked up to 120 min. It is interesting to note that **NCg** showed a slight increase in glucose levels 30 min after starting the test. However, the hypoglycemic effect exhibited by both groups was not related to the increase in plasma insulin. In fact, insulin levels remained unchanged throughout the test in the **NCg** group, while they decreased significantly from 30 min in the **HCg** group. Finally, the groups that received the main compound (**NJa** and **HJa**) behaved differently. A hypoglycemic effect was only perceived in the hyperglycemic group, which began 30 min after compound administration, being even more effective than glibenclamide. Regardless of this significant glucose reduction, plasma insulin tended to decrease after 30 min, returning to basal levels at 120 min. In the normoglycemic group, junceic acid prevented glucose levels from decreasing over time, promoting a slight but significant increase after 30 min. Surprisingly, insulin levels were significantly augmented, reaching a maximum rise of 250% at 90 min, in the normoglycemic rats that received the compound despite not showing a glucose-lowering effect.

All these findings suggest that the hypoglycemic effect exerted by EtOH-WCg and junceic acid in fasting is not related to the increase in plasma insulin, but rather to an additional action that may be involved in the direct or indirect improvement of insulin function. Since insulin levels remained unchanged or decreased, depending on the physiological state of the rats, the inhibitory effect on glucose production of both the extract and the compound could be replacing the function of insulin in controlling endogenous glucose production to maintain euglycemia or counteract hyperglycemia. However, the inhibitory effect on G6Pase exhibited by junceic acid could be so potent that, to avoid dangerous hypoglycemia in a healthy state, an increase in the expression of this enzyme would be promoted as a consequence of a regulatory feedback process. At the same time, a release of insulin to counteract the glucose overproduction may be stimulated. This must be confirmed in further studies.

#### 3.3.2 Postprandial condition

**FIGURE 6:**
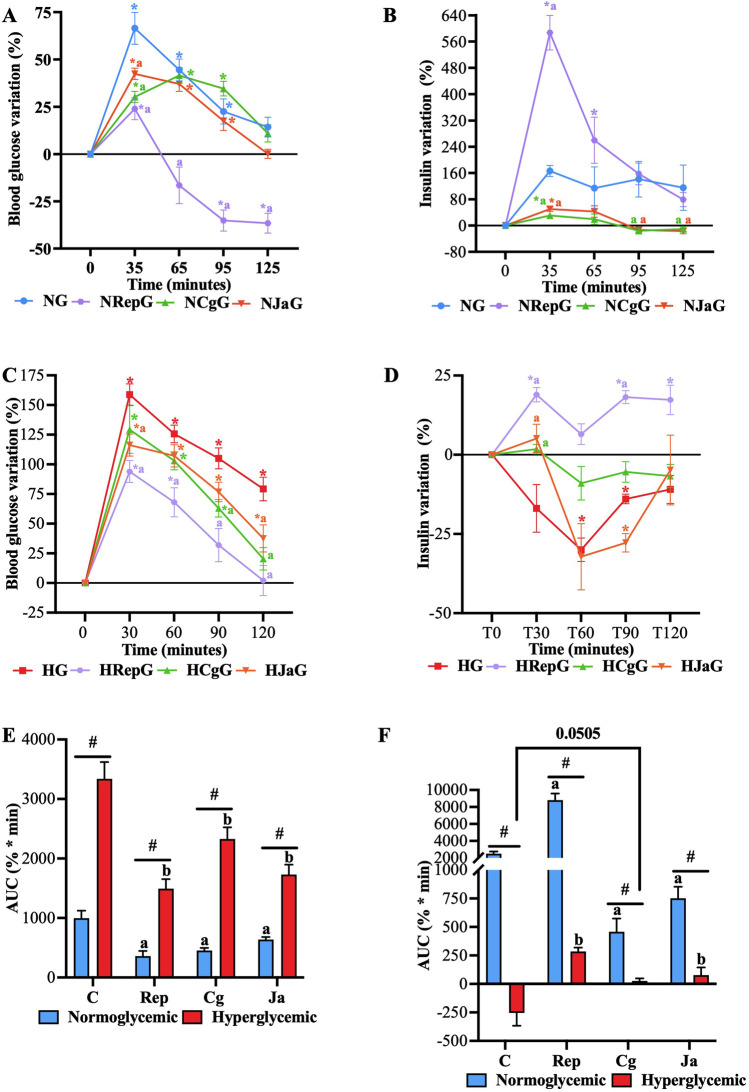
Effect of EtOH-WCg and junceic acid on insulin levels in postprandial state. **(A)** Blood glucose variation in normoglycemic groups; **(B)** Plasma insulin variation in normoglycemic groups; **(C)** Blood glucose variation in hyperglycemic groups; **(D)** Plasma insulin variation in hyperglycemic groups; **(E)** AUC of the hyperglycemic peak presented 30 min after de glucose load; **(F)** AUC of the hyperinsulinemic peak presented 30 min after de glucose load. *indicates statistically significant differences versus its initial time; *p* < 0.05, ^**#**^indicates statistically significant differences between normoglycemic and hyperglycemic groups per treatment; *p* < 0.05, ^**a**^indicates statistically significant differences versus **NG** (only within normoglycemic groups); *p* < 0.05. ^**b**^ indicates statistically significant differences versus **HG** (only within hyperglycemic groups); *p* < 0.05. **NG**, normoglycemic; **NRepG**, normoglycemic + repaglinide; **NCgG**, normoglycemic + EtOH-WCg; **NJaG**, normoglycemic + junceic acid isolated from ethanol-water extract of EtOH-WCg bark. **HG**, hyperglycemic; **HRepG**, hyperglycemic + repaglinide; **HCgG**, hyperglycemic + EtOH-WCg; **HJaG**, hyperglycemic + junceic acid isolated from EtOH-WCg.

To evaluate the effect on plasma insulin of both the extract and its main compound in the postprandial state, glucose tolerance tests were performed in normoglycemic and STZ-NA-hyperglycemic rats, where blood samples were obtained to quantify both glucose and insulin. As shown in Figures 6A, B, glucose load promoted a sustained glucose-stimulated insulin secretion (GSIS) response in healthy rats (**NG**), showing a maximum increase of 73% in blood glucose, which was the highest of all normoglycemic groups. Blood glucose subsequently returned to baseline levels. In hyperglycemic rats (**HG**), on the other hand, glucose load promoted a significant hyperglycemic peak of almost 175%, which did not return to basal levels (Figure 6C). This exacerbated postprandial hyperglycemia may be a product of the loss of the GSIS response, as seen in Figure 6D, where insulin levels significantly decreased compared with their baseline. Regarding repaglinide groups (**NRepG** and **HRepG**), it was observed that repaglinide significantly decreased the hyperglycemic peak that occurred after the glucose load in both normoglycemic and hyperglycemic rats (Figure 6E), effect that can be attributed to the increase in insulin levels promoted by the drug at 30 min (Figure 6F). In healthy rats, it increased insulin levels by more than 500%, exerting a marked hypoglycemic effect throughout the experiment, while glucose levels returned to baseline despite only increasing insulin by just under 25% in hyperglycemic rats.

In both normoglycemic and hyperglycemic rats administered with the extract (**NCgG** and **HCgG**), the hyperglycemic peak was significantly reduced after the glucose load. In healthy rats, this decrease was not related to an enhancement of GSIS; in fact, the extract significantly inhibited the GSIS response promoting a minor and delayed hyperglycemic peak. On the other hand, in hyperglycemic rats induced with STZ-NA, the extract reduced postprandial hyperglycemia by inhibiting the glucose peak at 30 min and promoting the return of glucose to basal levels at 120 min. This effect can be partially explained by the fact that the extract prevented the reduction of GSIS response, as seen in Figure 6D. Similar to the extract, junceic acid reduced the hyperglycemic peak and inhibited the GSIS response in healthy rats (**NJaG**), whose blood glucose returned to the baseline at the end of the experiment. When administered to hyperglycemic rats (**HJaG**), the compound could manage significantly postprandial hyperglycemia compared with the **HG**; however, although it did not completely prevent the drop in the GSIS response, it did avoid insulin levels from significantly declining compared with the control at 30 min after administration.

Despite not potentiating the GSIS response, the results overall indicated that both EtOH-WCg and junceic acid can manage postprandial hyperglycemia. It is important to emphasize that insulin release is not involved in the hypoglycemic mechanism that controls hyperglycemia in this metabolic state. However, the findings suggest that an improvement of insulin function may be involved as lower insulin levels were required to control blood glucose in healthy rats. In addition, a protective effect on pancreatic β-cell could also be exerted since GSIS response was slightly restored in hyperglycemic animals. Further experiments are encouraged to assess these hypotheses.

### 3.4 Effect on insulin sensitivity

#### 3.4.1 Insulin tolerance tests

**FIGURE 7:**
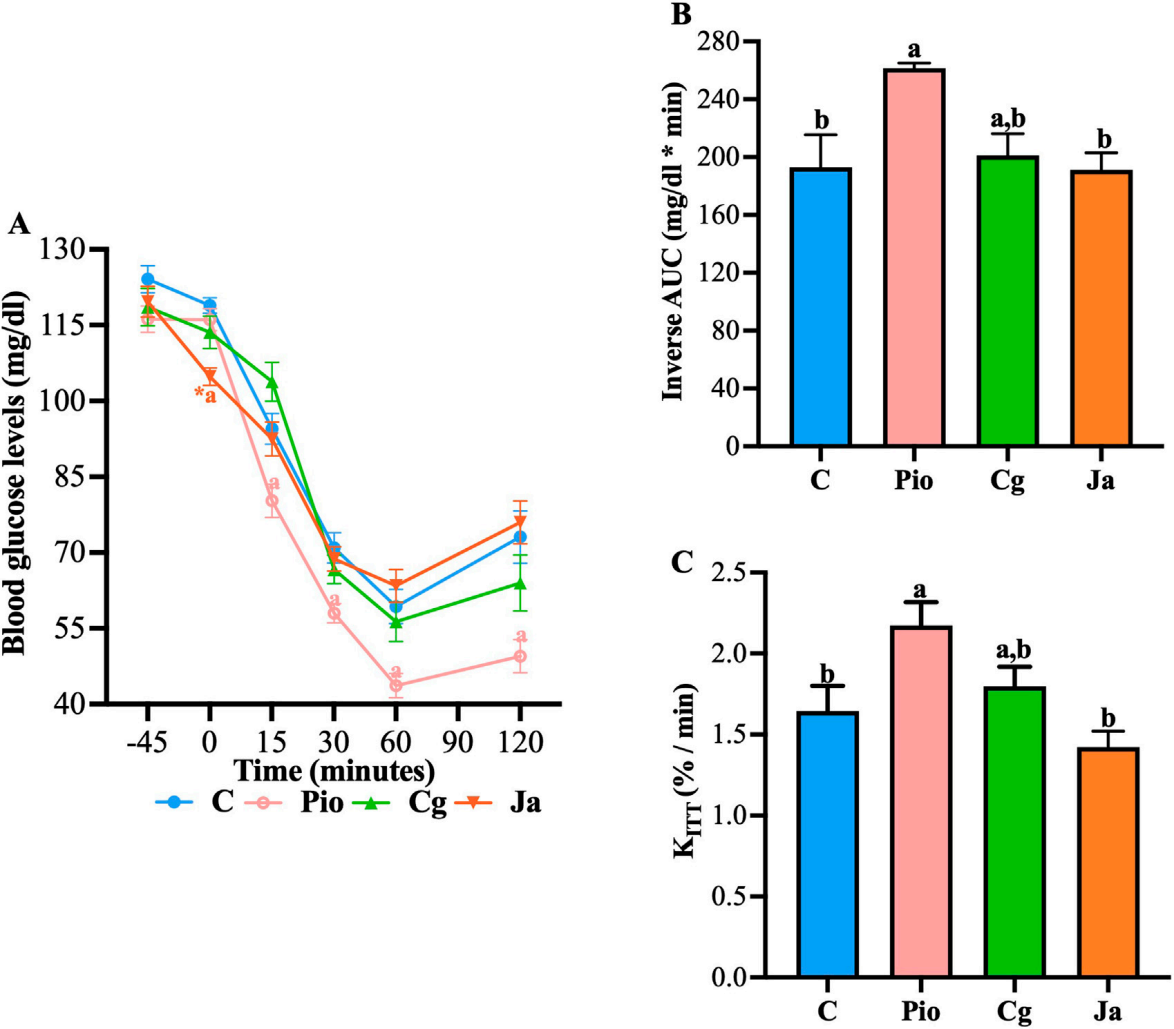
Effect of EtOH-WCg and junceic acid on insulin sensitivity. **(A)** Blood glucose levels in insulin tolerance tests. *indicates statistically significant differences between −45 min versus 0 min within the same group (*t*-test; *p* < 0.05), ^**a**^indicates statistically significant differences versus **C**; *p* < 0.05. **(B)** Inverse AUC values. Different letters over the bars indicate statistically significant differences (a > b); *p* < 0.05. **(C)** K_ITT_ values. Different letters over the bars indicate statistically significant differences (a > b); *p* < 0.05. The values represent the mean ± SEM (n = 6). **C**, control; **Pio**, pioglitazone; **Cg**, EtOH-WCg; **Ja**, junceic acid isolated from EtOH-WCg.

As previously suggested, both EtOH-WCg and junceic acid could promote better insulin sensitivity. To determine whether both directly enhance insulin function, insulin tolerance tests were carried out in healthy rats with 4 h of fasting. In these glucose time curves, insulin injection stimulated a pronounced decrease in glucose levels that was counter regulated after 60 min, where a slight increase was observed in control (**C**). The group administered with pioglitazone (**Pio**), used as control drug in this test, exacerbated the glucose-lowering effect of insulin from the first 15 min after insulin administration when compared against **C**. This enhancing effect was maintained throughout the 120 min of the test. On the other hand, neither the extract nor the junceic acid potentiated the effect of insulin, as no significant differences were observed at any time compared with **C**. However, the group administered with junceic acid (**Ja**) showed a significant hypoglycemic effect before insulin injection (Figure 7A). When performing the inverse AUC and K_ITT_ analyzes, it was observed that pioglitazone eliminated more glucose throughout the experiment at a higher removal rate than **C**, while the EtOH-WCg had an intermediate behavior between **C** and **Pio** and junceic acid had the same effect as that shown by **C** (Figures 7B, C). Therefore, it was concluded that neither the extract nor the compound participates in the improvement of the glucose-lowering response of insulin.

#### 3.4.2 Concentration-response inhibition assays of PTP-1B

**FIGURE 8:**
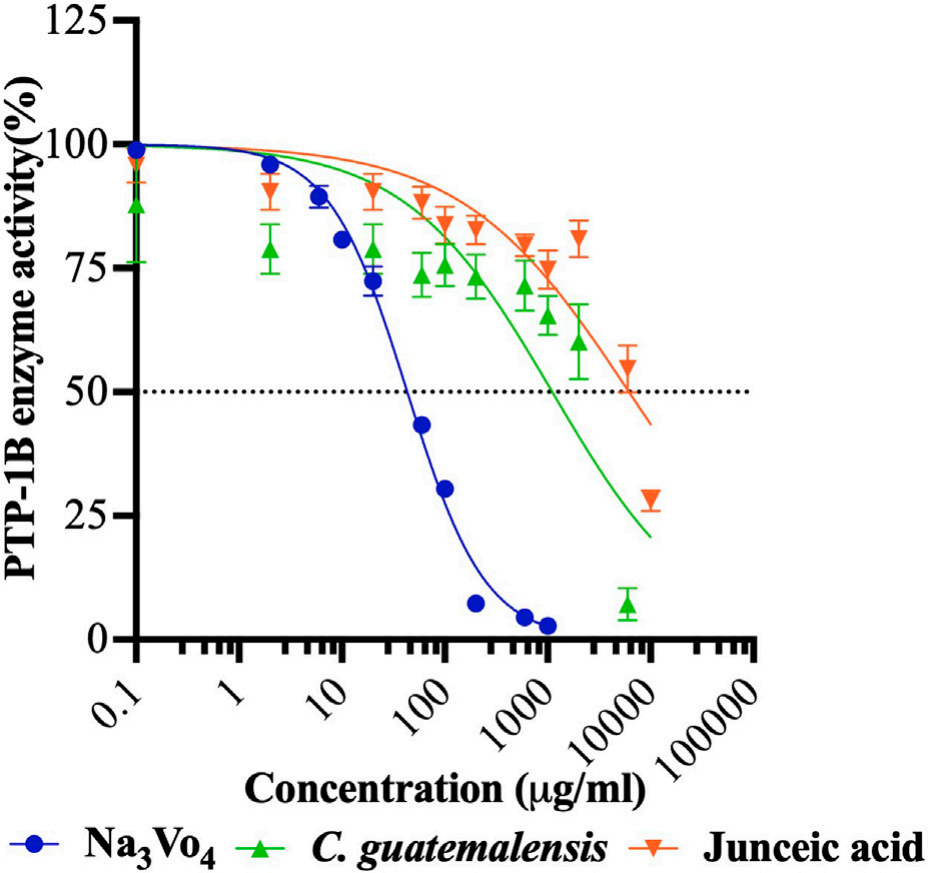
Concentration-response inhibition curves of EtOH-WCg and junceic acid on enzyme activity of PTP-1B. Na_3_VO_4_ was used as positive control. Each point represents the mean ± SEM of three independent assays in triplicate.

Inhibition of PTP-1B activity has been identified as an attractive target for natural compounds to improve insulin function since it is involved directly in termination of insulin signalling. As shown in Figure 8; Table 2, both extract and compound inhibited the enzyme at low potency, exhibiting IC_50_ values 26 and 143 times higher than the control, respectively. However, EtOH-WCg decreased enzyme activity effectively by 93%, which could explain the tendency to enhance the insulin effect *in vivo*. The results of *in vitro* assays support the findings obtained in insulin tolerance tests.

**TABLE 2:** *In vitro* assessment of EtOH-WCg and junceic acid on enzyme activity of PTP-1B.

Protein tyrosine phosphatase 1B (PTP-1B)		
Sample	Inhibition %	IC50 ± SEM (µg/mL)
Na_3_VO_4_ ^a^	97	43.2 ± 1.5
EtOH-WCg	93	1,109 ± 246.5
Junceic acid	72	6,170 ± 1,339.8

^a^ Positive control.

## 4 Discussion

The use of natural remedies to treat diseases is common worldwide. Medicinal plants can be utilized in combination with, or instead of prescribed, drugs (Cruz and Andrade-Cetto, 2015). This use is particularly common in the treatment of metabolic diseases such as diabetes, since the classic medications used to manage hyperglycemia, such as metformin, glibenclamide, or acarbose, have low adherence due to uncomfortable side effects. In this context, evaluating the pharmacological effects of these traditional preparations is imperative to provide more information on their possible therapeutic and/or toxicological activities. Through an ethnopharmacological approach, medicinal plants with effective action mechanisms have been described in the literature (Heinrich and Gibbons, 2001; Patwardhan, 2005; Andrade-Cetto and Heinrich, 2011). The advantage of mixtures of compounds from natural sources is that synergistic effects can be produced through the activation or inhibition of different pathways (Caesar and Cech, 2019), which can be very useful in the treatment of multifactorial diseases such as T2D.

The *Croton* genus includes species that may be promising sources of compounds with different therapeutic mechanisms of actions to alleviate hyperglycemia. However, the mechanisms of action of most species with reported hypoglycemic and antihyperglycemic effects remain unknown (Espinoza-Hernández et al., 2023). With the aim of improving our knowledge of the biological actions of the genus, studies to elucidate the main mechanisms of *C. guatemalensis*, as well as its main compound junceic acid, were proposed. To delay diabetic complications and achieve effective glycemic control, fasting and postprandial hyperglycemia should be managed to maintain glucose variations within a specific range (ElSayed et al., 2024b). Therefore, a polypharmacological approach could be more beneficial as assorted mechanisms that promote hyperglycemia could be modulated in both states (Reddy and Zhang, 2013; Barre and Mizier-Barre, 2020). In this sense, medicinal plants used to treat diabetes, such as *C. guatemalensis*, are usually ingested throughout the day, so glycemic modulation of several targets in both states could occur (Cruz and Andrade-Cetto, 2015).

In the current investigation, we demonstrated that both EtOH-WCg and junceic acid could alleviate fasting hyperglycemia through the inhibition of hepatic glucose production. Specifically, the activity of two regulatory enzymes was directly inhibited. To the best of our knowledge, this is the first report on the regulation of this mechanism by extracts of the *Croton* genus. Hence, these findings demonstrate that *Croton* species may be a source of potent enzymatic modulators of this pathway, as observed in the case of junceic acid, and partially explain the traditional use of EtOH-WCg as “agua de uso.” According to the literature, clerodane diterpenoids exhibit a wide range of biological actions, including insect anti-feeding and insecticidal properties; opioid receptor modulatory activity; and antitumor, antifungal, nerve growth factor-potentiating, antibiotic, anti-peptic ulcer, antiplasmodial, hypoglycemic, hypolipidemic, and anti-thrombin inhibition activities (Li et al., 2016). Therefore, the inhibition of enzymes that participate in endogenous glucose production is also a new mechanism of action for this group of metabolites.

Although both EtOH-WCg and junceic acid were shown to directly inhibit G6Pase and FBPase activities, evidence suggests that their impacts on the negative regulation of hepatic glucose production are different. The finding that the extract inhibited both enzymes effectively *in vitro* resulted in a better antihyperglycemic effect in the pyruvate tolerance tests (Figure 3). In this study, gluconeogenesis was the major pathway assessed as the rats were fasted for 18 h. Consequently, the main glucose-producing pathway inhibited by this extract is likely the pathway by which glucose is synthesized *de novo* from other noncarbohydrate substrates. In accordance with this approach, the blood glucose levels of both healthy and hyperglycemic animals fasted overnight were reduced after extract administration. Unlike humans, rodents become completely dependent on gluconeogenesis after overnight fasting, as a rapid catabolic state was promoted, in which glucose stores are drastically mobilized and liver glycogen stores are depleted (Edgerton et al., 2017; Benedé-Ubieto et al., 2020). Therefore, gluconeogenesis plays a key role in maintaining blood glucose levels in overnight-fasted rats.

**FIGURE 9:**
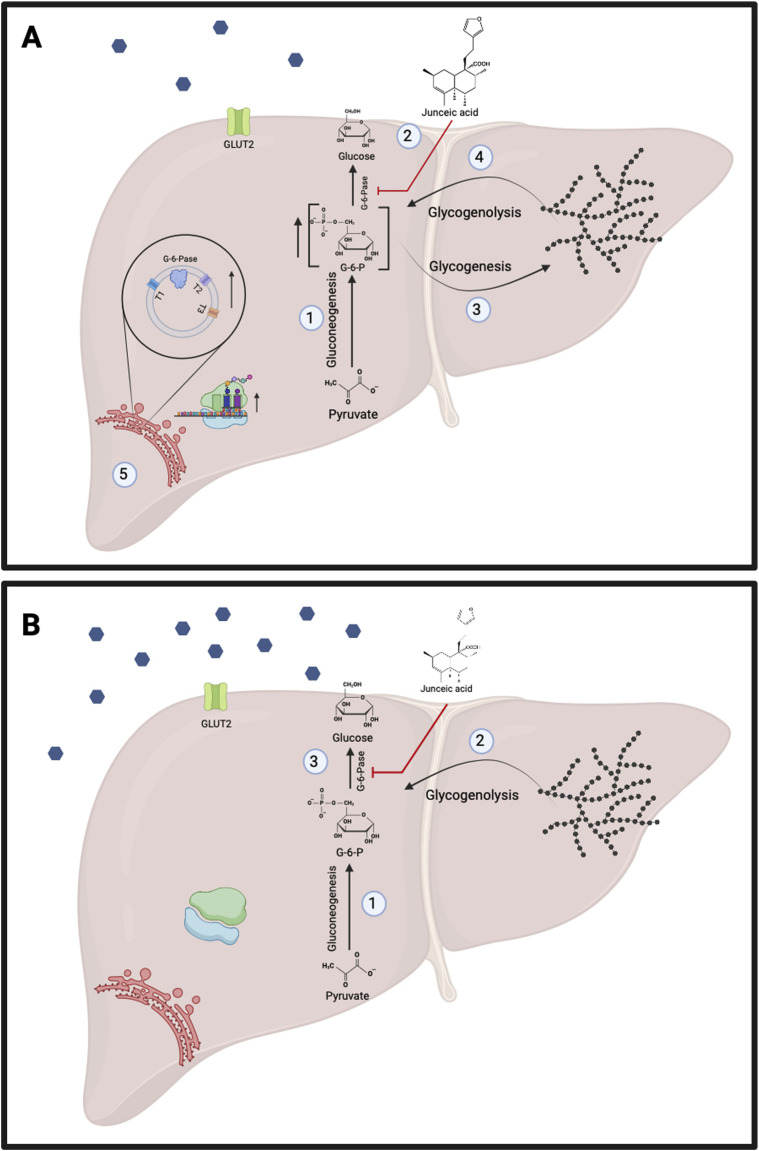
Proposed effects of junceic acid in the fasting state. **(A)** Healthy rats. 1.- Concentration of glucose-6-phosphate increases through the activation of gluconeogenesis. 2.- Junceic acid strongly inhibits activity of glucose-6-phosphatase. 3.- Due to the inhibition of glucose-6-phosphatase, glucose levels decline, resulting in the activation of glycogenolysis. 4.- Gluconeogenesis and glycogenolysis lead to the accumulation of glucose-6-phosphate which activates glycogenesis. 5.- The potent inhibition of glucose-6-phosphatase by junceic acid raises the expression of this enzyme and GLUT2 with the potential objective of regulating the export of glucose into the bloodstream. **(B) Hyperglycemic rats**. 1.- Gluconeogenesis is overactive increasing concentration of glucose-6-phosphate. 2.- Glycogenolysis is also active due to deficient insulin levels. Both glucose-producing pathways contribute to fasting hyperglycemia. 3.- Junceic acid inhibits glucose-6-phosphatase, diminishing fasting hyperglycemia. Created with BioRender.com.

On the other hand, the potent negative effect of junceic acid on G6Pase activity had different effects on endogenous glucose production depending on the physiological state of animals (Figure 9). In previous studies performed with healthy starved rats and isolated hepatocytes, inhibition of G6Pase led to the accumulation of G6P without altering gluconeogenic flux. G6P is an allosteric activator of glycogen synthase, so glycogen content increases under these conditions. Since the inhibition of G6Pase, which is an inhibitor of glycogen phosphorylase, also decreases glucose levels, the activity of this enzyme remained high. Consequently, the continuous degradation and synthesis of glycogen contributes to G6P homeostasis. Furthermore, the mRNA levels of GLUT2 and G6P translocase increased, indicating that they may play an important role in regulating the intracellular concentration of G6P through the export of cytosolic glucose into the bloodstream (Herling et al., 1998; van Dijk et al., 2001). As a result, potent inhibition by junceic acid in overnight-fasted healthy rats induced the intracellular accumulation of G6P, thereby strongly stimulating glycogen synthesis and degradation, which resulted in the continuous release of glucose into the bloodstream as a mechanism to regulate homeostasis (Figure 5A). Simultaneously, β cells sensed this increase in glucose levels and secreted a greater amount of insulin to maintain euglycemia (Figure 5B). In this sense, importantly, junceic acid seems not to be crucial for reducing gluconeogenic flux because it does not potently inhibit FBPase activity.

As mentioned above, the glucose levels of a rodent that is fasted overnight are primarily maintained by gluconeogenesis. Hence, glycogenolysis preserves euglycemia during the post-absorptive period (after 4 h of fasting). This effect could explain the opposing effects of junceic acid in healthy rats in two different experiments: a 12-h fast was used in the *in vivo* test to assess the role of plasma insulin, and a 4-h fast was utilized in the *in vivo* insulin sensitivity test. Because gluconeogenesis is the only pathway activated after overnight fasting, inhibition of G6Pase by junceic acid triggers the regulatory feedback process explained above. On the other hand, this response is not promoted when glycogenolysis is the only contributor to endogenous glucose production; thus, a sustained hypoglycemic effect was observed.

In the pathological state of hyperglycemia generated by STZ, overexpression of G6Pase and increased G6Pase activity have been reported (Marcolongo et al., 2013; Govindarajan et al., 2021), leading to the release of substantial amounts of glucose from gluconeogenesis or glycogenolysis into the bloodstream, as observed in the control hyperglycemic group, where blood glucose levels increased over time (Figure 5C). Therefore, the potent decrease in G6Pase activity induced by junceic acid resulted in a more pronounced decrease in glucose levels in 12-h fasted hyperglycemic rats. This decrease was even more marked than that observed for the extract and glibenclamide.

In both healthy and hyperglycemic rats, the administration of extract did not stimulate an increase in insulin levels, ruling out insulin secretion as one of the mechanisms contributing to the hypoglycemic effect when consumed orally. Conversely, administration of the extract did not affect insulin variation in healthy rats and promoted a significant decrease in insulin levels in hyperglycemic rats (Figure 5D). This result can be explained by the fact that insulin secretion adjusts to the metabolic demand of insulin-sensitive tissues; thus, any change that alters insulin sensitivity would result in greater or less secretion (Dimitriadis et al., 2021). Hence, EtOH-WCg may increase insulin sensitivity because less insulin is required to ameliorate hyperglycemia. Nevertheless, the effect of the extract on insulin sensitivity was not related to a direct enhancement of insulin function, as shown by insulin tolerance tests and PTP-1B inhibition assays (Figures 7, 8); therefore, these results must be induced by another insulin-independent mechanism, such as direct inhibition of endogenous glucose production or activation of the AMP-activated protein kinase (AMPK) pathway. These results agree with previous findings that show that the administration of phytomodulatory proteins from the latex of *Calotropis procera* (Aiton) W.T. Aiton (Apocynaceae) decreased blood glucose levels without altering insulin concentrations in healthy rats by promoting the inhibition of gluconeogenesis via AMPK (de Oliveira et al., 2019). Likewise, the adipose-tissue-derived factor Acrp30 was shown to stimulate a sustained hypoglycemic effect unrelated to an increase in plasma insulin in healthy, insulin-resistant, hyperglycemic mice through the suppression of hepatic glucose production (Berg et al., 2001). This phenomenon was partially replicated by junceic acid under hyperglycemic conditions, although insulin levels returned to baseline at the end of the experiment, suggesting a less potent noninsulin-dependent sensitizing effect.

**FIGURE 10:**
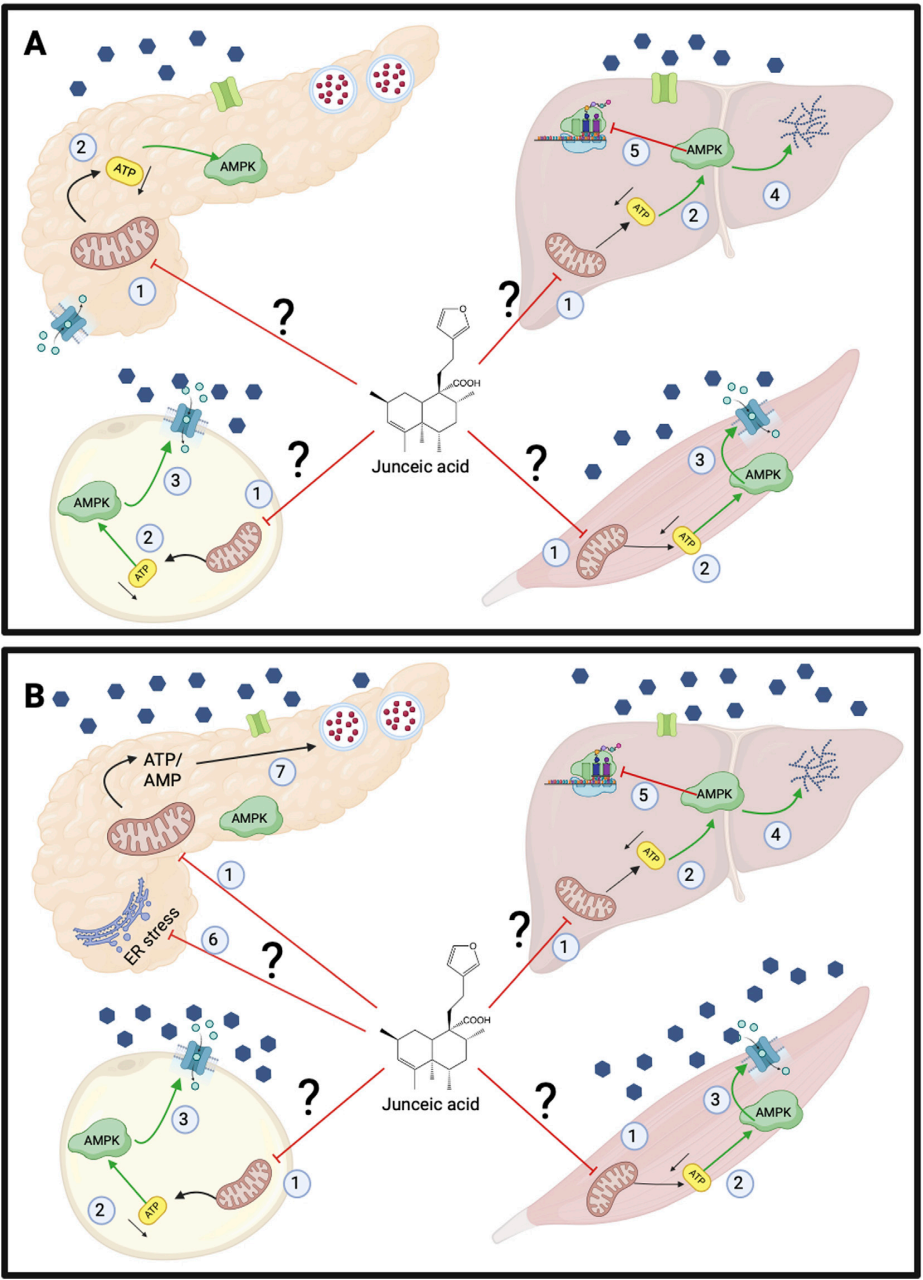
Proposed effects of junceic acid in the postprandial state. **(A)** Healthy rats. 1.- Junceic acid may inhibit the production of ATP by mitochondria. 2.- Low levels of ATP activates AMPK. Specifically, in pancreatic β cell, the decrease in ATP blocks insulin secretion. 3.- AMPK may promote GLUT4 translocation in muscle and adipose tissue. 4.- In liver, AMPK could promote glycogen production. 5.- AMPK also inhibits expression of gluconeogenic enzymes. **(B)** Hyperglycemic rats. 1.- Junceic acid may inhibit the production of ATP by mitochondria. 2.- Low levels of ATP activates AMPK. 3.- AMPK promotes GLUT4 translocation in muscle and adipose tissue. 4.- In liver, AMPK may promote glycogen production. 5.- AMPK also inhibits expression of gluconeogenic enzymes. 6.- Junceic acid may promote metabolic deceleration in pancreatic β cell by reducing endoplasmic reticulum stress and 7.- restoring ATP/AMP balance which leads to recovery of insulin secretion. Created with BioRender.com.

Postprandially, both extract and junceic acid decreased the hyperglycemic peak at 30 min, thereby promoting improved glucose tolerance throughout the experiment in both healthy and hyperglycemic rats without exacerbating the GSIS response (Figure 6). In fact, suppression of the GSIS response was observed in healthy rats, while partial recovery of this response was shown in hyperglycemic rats (Figure 10). In addition to exerting a potential non-insulin-dependent sensitizing effect on the liver or peripheral tissues that may increase energy expenditure, both EtOH-WCg and junceic acid may directly inhibit insulin secretion through the stimulation of AMPK in β-cells under physiological conditions, as shown in previous studies with pioglitazone, metformin, and berberine (Lamontagne et al., 2009; 2013; Bai et al., 2018; Huang et al., 2021). Although there is conflicting evidence on the effect of AMPK activation on insulin secretion (Szkudelski and Szkudelska, 2019), EtOH-WCg and junceic acid promoted a decrease in the GSIS response in this study; therefore, the pharmacological impact of both the extract and junceic acid on β-cells should be assessed to establish the possible link between AMPK and insulin secretion in further *in vivo* and *in vitro* experiments.

On the other hand, both the extract and compound may protect β-cells under glucolipotoxic conditions via AMPK activation, which will promote metabolic deceleration by decreasing the level of oxidative stress and endoplasmic reticulum stress and preventing excessive lipid accumulation and cellular dysfunction (Andrikopoulos, 2010; Szkudelski and Szkudelska, 2019; Huang et al., 2021; Karunakaran et al., 2021). All these actions can help reverse the alteration of the ATP/ADP ratio, recover the GSIS response, and reduce β-cell dysfunction (Kim et al., 2016). However, the extract exerted a better protective effect on β-cells than junceic acid, which could probably be due to the presence of other components that, together, may exhibit a synergistic effect (Figure 6D).

Other *Croton* species and their isolated compounds, such as 3β-hydroxyhop-22 (29)ene, fern-9 (11)-ene-2α,3β-diol, and 2α,3β,23-trihydroxyolean-12-ene from *C. heterodoxus* Baill. (Gomes Castro et al., 2015; 2021; da Luz et al., 2016), and *C. klotzschianus* (Wight) Thwaites (Govindarajan et al., 2008), have been shown to improve oral glucose tolerance by exerting antihyperglycemic effects associated with an increase in insulin secretion. However, in the current research, EtOH-WCg was shown to ameliorate postprandial hyperglycemia by exerting the opposite effect in healthy rats, that is, by reducing insulin secretion and partially restoring the GSIS response in hyperglycemic rats. This finding is relevant because current pharmacological therapies seek to protect β-cells from exhaustion related to continuous insulin secretion and caused by the glucolipotoxicity characteristic of diabetes (Robertson, 2009; Huang et al., 2021). Hence, this species and its active phytochemicals may improve the diabetic status of patients who traditionally consume it.

Overall, the present work provides evidence about the polypharmacological effects of *C. guatemalensis* and its main compound, junceic acid, in the fasting and postprandial states (Figures 9, 10), thereby shedding light on its therapeutic targets for diabetes management. Further work should aim to verify that *C. guatemalensis* and junceic acid are AMPK activators and characterize their tissue-specific impact on fasting and postprandial states in both healthy and insulin-resistant models. Likewise, their long-term impact must be assessed, especially in relation to insulin resistance, efficacy parameters such as glycated hemoglobin, oxidant and inflammatory stress markers, as well as lipid profiles.

## 5 Conclusion

In the present study, the hypoglycemic effects of *C. guatemalensis*, a traditional species used to treat T2D in Guatemala, were characterized. This species managed fasting hyperglycemia by decreasing hepatic glucose production, specifically the activity of the rate-limiting enzymes G6Pase and FBPase and exerting a noninsulin-dependent sensitizing effect. Furthermore, the extract controlled postprandial hyperglycemia without potentiating the GSIS response, probably through the activation of AMPK in β−cells, which, under glucotoxic conditions, could exert a protective effect. On the other hand, although junceic acid, the main compound in the EtOH-WCg, exhibited effects similar to those of the complete extract, the inhibition of hepatic glucose production was its main pharmacological action, as it was demonstrated to be a potent activator of G6Pase. Future work to characterize the implications of the long-term effects of the complete extract and junceic acid, as well as to test the effect of both on AMPK activation, should be performed.

## Data Availability

The raw data supporting the conclusions of this article will be made available by the authors, without undue reservation.
